# Mobile phone dependence among Chinese university students: the contribution of irrational beliefs and solitude

**DOI:** 10.3389/fpsyg.2024.1453961

**Published:** 2024-10-01

**Authors:** Xiaoxue Kuang, Kerry John Kennedy, Hui Li

**Affiliations:** ^1^Department of Education, School of Education (Normal School), Dongguan University of Technology, Dongguan, China; ^2^Department of Curriculum and Instruction, The Education University of Hong Kong, Ting Kok, Hong Kong SAR, China; ^3^Department of Education and Curriculum Studies, University of Johannesburg, Johannesburg, South Africa; ^4^School of Education, Guangzhou University, Guangzhou, China

**Keywords:** solitude, irrational beliefs, mobile phone dependence, university students, psychosocial environments

## Abstract

This research aimed to explore the impact of selected psychological variables on mobile phone dependence among Chinese university students. Specifically, the study focused on the relationship between solitude and irrational beliefs on mobile phone dependence. The study included 2,888 university students from Guangdong, Southern China, and found that both irrational beliefs and solitude had significant direct effects on mobile phone dependence. The study also revealed that solitude mediated the relationship between irrational beliefs and mobile phone dependence. The results of this study indicate that psychosocial environments, including feelings of solitude and irrational beliefs, can influence mobile phone dependence among undergraduate students. Therefore, it is important to pay attention to these factors and their potential impact on student behavior, particularly when it comes to academic growth and development. Further research in this area may help identify effective strategies to address mobile phone dependence among university students and promote healthier psychosocial environments.

## Introduction

With the rapid development of information technology and 5G networks, the number of mobile phone users has substantially increased over the last several years. According to the Global Market Report (Newzoo, 2021), the number of mobile phone users worldwide will reach to a total of 4.1 billion by 2023, and over a quarter of these will be Chinese. There are approximately 10.5 billion Chinese internet users as of June 2022 and 99.6% of them used a mobile phone (China Internet Network Information Center, 2022). Users spent an average of 29.5 h online every week. Among Chinese internet users, 13.5% are 10–19 years old. These data show that students in China are increasingly spending much of their time with their mobile phones. Specially, students entering university with less supervision of their family, are more likely to indulge themselves using mobile phones (Bian and Leung, 2015). Such phones have brought enormous convenience to people including daily connections, working, online shopping, games and so on. The previous studies have indicated that over 30% of adolescents and adults exhibit problematic mobile phone use in China (Gao et al., 2022; Luk et al., 2018). And it has been shown that excessive mobile phone use has the potential to cause significant negative consequences (Soror et al., 2015).

Mobile phone dependence has been identified as a major issue among Chinese university students (Liu et al., 2021). Sometimes it referred to as mobile phone addiction (Chen et al., 2021; Yang et al., 2020; Sun et al., 2019; Sahu et al., 2019; Ivanova et al., 2020). At other times, it is referred to nomophobia (Tams et al., 2018; Bhattacharya et al., 2019). In all cases, dependence on a mobile phone has been commonly defined as an uncontrollable overuse of a mobile phone resulting in negative consequences influencing the daily functioning of the user. Given the serious consequences and high prevalence of mobile phone dependence, research on its antecedents and psychological processes is necessary to provide theoretical guidance for prevention and intervention efforts.

## Literature review

### Mobile phone dependence

Choosing a definition of mobile phone dependence/addiction etc. needs to be done carefully. Liu et al. (2021) distinguished between the concept of mobile phone dependence, mobile phone addiction and problematic mobile phone use. Mobile phone addiction, if seen as a psychiatric diagnostic term signaling uncontrolled use of a mobile phone, might not meet the criteria of additional symptoms. The concept of problematic mobile phone use was seen to be too broad for this study as it contains “not only mobile phone addiction/dependence but also other problematic behavior of mobile phone use” (Liu et al., 2021, p. 5134). Thus, this study adopted the definition of Liu et al.’s (2021) who used mobile phone dependence defined as “the excessive and uncontrolled use of mobile phones in spite of the significant negative consequences in social, behavior, and affective aspects” (p. 5134). Thus, the construct, mobile phone dependence, was used in this study.

Mobile phone dependence has been associated with a range of physical and mental health issues. Existing studies have shown that mobile phone dependence was related to lower psychological well-being (Kumcagiz and Gunduz, 2016), health problem (Wacks and Weinstein, 2021), lower social skills and emotional intelligence (Scott et al., 2017), lower self-esteem (Hong et al., 2012), unhealthy behavior (Kim and Han, 2020), academic performance (Troll et al., 2021), impulsivity, and sleep quality, depression and anxiety (Elhai et al., 2019; Li et al., 2020; Wang et al., 2022). Thus, to better understand the reason for students’ mobile phone dependence, it is important to explore the factors that might be associated with such dependence and the underlying psychosocial mechanisms.

### Irrational beliefs and mobile phone dependence

Previous studies have demonstrated that alexithymia (Hao et al., 2019; Mei et al., 2018), early maladaptive schema (Arpaci, 2021), mental disorder (Alavi et al., 2020; Sun et al., 2022), shyness, attachment anxiety and self-control (Han et al., 2017), insecure attachment (Zhang et al., 2022), loneliness (Liu et al., 2021; Tan et al., 2013) and parent-child relationship (Niu et al., 2020) directly predicted mobile phone dependence or mobile addiction. In addition, gender also predicted mobile phone dependence (Busch and McCarthy, 2021). However, few studies have paid attention to the effect of irrational beliefs which are important factors that can promote a better understanding of human behavior.

Irrational beliefs have been defined as unrealistic “reasoning processes by which external events are interpreted and through which emotional distress is mediated” (Koopmans et al., 1994, p. 15). It was first proposed by Ellis (1962) under the framework of rational emotive behavior therapy (REBT) and has played a central role in cognitive theory and therapy. Initially, the framework identified 11 types of irrational beliefs which Ellis believed could cause emotional disturbance for individuals. Ellis and Dryden (1987) modified these to three irrational beliefs that are now commonly used. According to REBT, it is people’s beliefs that underpin their emotional consequences (Ellis, 1994). Thus, a person’s behavior may depend on the beliefs they hold to explain the event.

Irrational beliefs linked to individuals’ dysfunctional responses to various situations (Bridges and Harnish, 2010), and several scales had been developed to measure these beliefs. Research suggested that irrational beliefs contribute to maladaptive behaviors like withdrawal, disordered eating, and alcohol misuse (Turner et al., 2022; Nolan and Jenkins, 2019; Tan et al., 2013). Moreover, irrational beliefs had been connected to burnout (Turner and Moore, 2016). Additionally, irrational beliefs were correlated with negative emotions, such as anxiety and depression (Baytemir, 2022; Zhang et al., 2021), and various disorders, including alexithymia, mental disorders (Zakiei et al., 2021), food addiction (Nolan and Jenkins, 2019), phobic anxiety (Thyer et al., 1987), and avoidance behavior (Warren et al., 1989). Sabanci and Çekiç (2019) also demonstrated that irrational beliefs predicted cyberbullying. However, the relationship between irrational beliefs and mobile phone dependence has yet to be explored.

### Solitude and mobile phone dependence

Solitude is a state of psychological feeling that is not equated with isolation or loneliness. It is a complicated concept which not only means “physical separateness from others” or being alone, but also means “a mental space characterized by inner focus” (Weinstein et al., 2022, p. 8). There are positive and negative aspects of solitude. Positive solitude reflects one’s ability to enjoy solitary experiences without feeling loneliness. Positive solitude might yield great benefit toward personal spiritual growth, distilling clarity and creativity, fostering moral courage, achieving self-actualization, and maintaining good health (Gordon, 2022). Negative solitude includes loneliness, meaning the state of absence of other people or isolation from others (Palgi et al., 2021). Chen et al. (2012) further proposed that solitude behavior can be classified into four types, each driven by different reasons or motivations: positive solitude, eccentricity, social avoidance, and loneliness.

The psychological significance of these solitude behavior varies. Positive solitude has a beneficial impact on individual psychological development, whereas the latter three typically reflect the negative aspects of an individual’s psychological characteristics. Among them, loneliness was the most studied and is defined as an unpleasant feeling that occurs when people perceive their network of social relationships to be quantitatively or qualitatively deficient (Perlman and Peplau, 1981). According to Maslow’s (1943) hierarchy of needs, a sense of belonging is one of a human’s most fundamental needs. If it is not met, an individual may feel anxious and tense and might tend to misbehavior. Research has found that solitude has been associated with anxiety and depression (Bermingham et al., 2021; Boursier et al., 2020; Cauberghe et al., 2021; Okruszek et al., 2020; Danneel et al., 2019). Studies have also shown that loneliness can be related to student's inclination for traumatic use of a mobile phone (Narimani and Ranjbar, 2016), mobile phone addiction (Zhang et al., 2020), nomophobia (Lu et al., 2022) and mobile phone dependence (Li et al., 2021). Loneliness was also found to be a mediator of the relationship between “childhood maltreatment” and mobile phone addiction (Ma et al., 2020). While preference for solitude was also found to be related to mobile phone addiction (Chen et al., 2021). Solitude, no matter positive or negative, is related to mobile phone use dependence.

### Mediating effect of solitude for irrational beliefs and mobile phone dependence

According to Ellis, REBT is based on the assumption that the tendency to make “devout, absolutistic evaluations” of life events is central to emotional disturbance (Robertson, 2018). These value judgments are often framed in dogmatic terms such as “must,” “should,” “have to,” “got to,” and “ought to,” which are considered unconditional imperatives (Dryden and Ellis, 2001, p. 301). These are termed “irrational beliefs” because they embody rigid and unrealistic demands that conflict with the goals of enlightened self-interest (Robertson, 2018). Thoughts and patterns of inaccurate causal attribution are considered primary factors, not merely triggers or maintainers of maladaptive emotions and behavior (Sheldon, 2011). Therefore, the higher the frequency of irrational beliefs, the more likely individuals are to experience social avoidance and negative emotional states such as anxiety and loneliness (Hyland et al., 2019). Some research further indicated that has also suggested that students who hold irrational beliefs—such as unrealistic expectations, self-downing, catastrophizing beliefs, or rigid thinking—are more likely to experience loneliness as a negative aspect of solitude (Hyland et al., 2019; Hoglund and Collison, 1989). These irrational beliefs may contribute to negative emotional states, making it more difficult for individuals to form or maintain healthy social connections, thereby increasing feelings of isolation. These negative emotions may increase the likelihood of spending excessive time on mobile phones as a way to escape from the real world (Lian et al., 2023). Given the potential effects of irrational beliefs on loneliness, and the impact of loneliness on mobile phone dependence, the key question remains whether loneliness can serve as a mediator in the relationship between irrational beliefs and mobile phone dependence.

**Figure 1:**
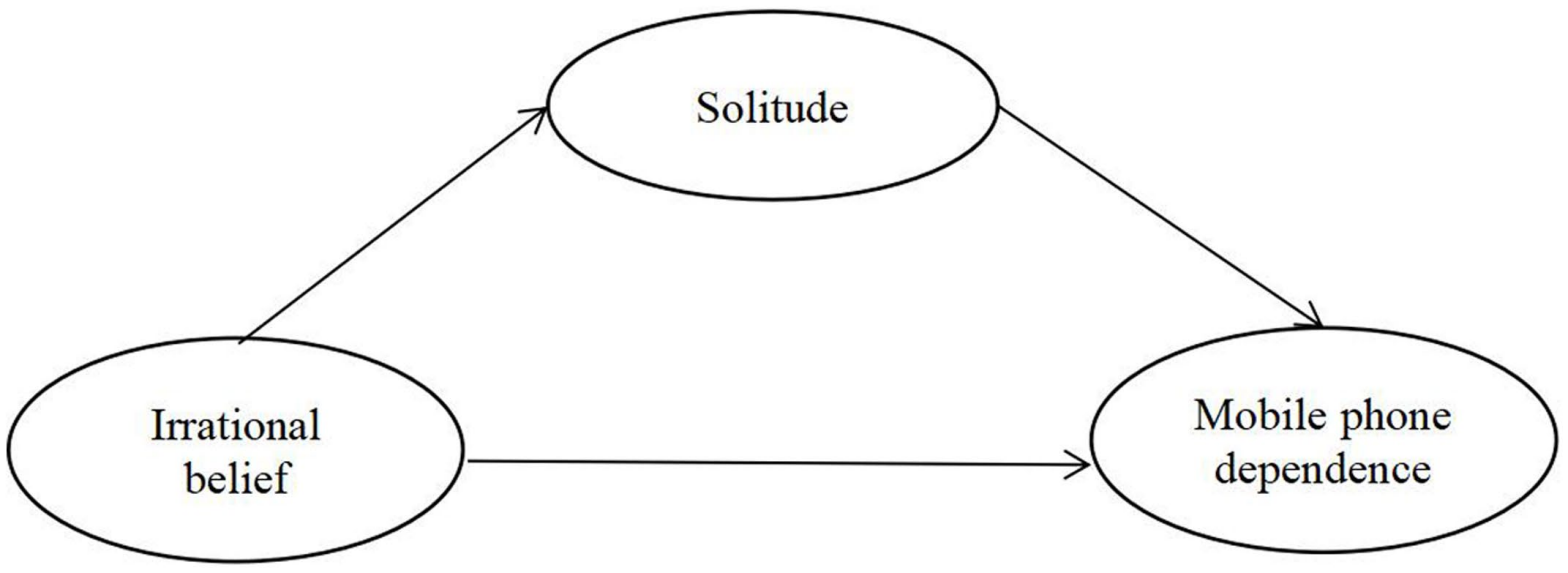
Theoretical model in this study.

Based on the aforementioned literature review, this study proposes a hypothetical model in which irrational beliefs and solitude are posited as predictors of mobile phone dependence, with solitude acting as a mediator. The proposed model is illustrated in Figure 1. The study will explore the following three research hypotheses:

*Hypothesis 1*: Irrational beliefs and solitude are associated with mobile phone dependence.

*Hypothesis 2*: Irrational beliefs are associated with solitude.

*Hypothesis 3*: Solitude mediates the relationship between irrational beliefs and mobile phone dependence.

## Method

### Participants

A total of 2,888 respondents, aged 17–19, voluntarily participated in this study. The sample included 69.5% male students and 30.5% female students. 7.4% students were from single-parent family 49.9% of the students lived in town, 50.1% of them lived in countryside. 20.7% of the students were from families with only one child.

### Procedure

Participants completed a self-report questionnaire that assessed mobile phone dependence, irrational beliefs, solitude, and collected background information. Data were collected online using the school APP platform. Prior to participation, participants were informed about the purpose of the study and provided informed consent. Following data collection, confirmatory factor analysis (CFA) was conducted to examine the factor structure of the scale. Analyses were conducted using SPSS 27.0 and Mplus 8.0, following standard procedures for descriptive statistics, reliability testing, and structural equation modeling.

### Measures

#### Irrational beliefs

Irrational beliefs were measured by 11 items derived from the statements of Ellis’ irrational beliefs’ framework (Newmark et al., 1973; Ellis and Dryden, 1987). The 11 statements had been translated into Chinese (Zhao, 2016). Dichotomous response categories (1 = yes, 2 = no) were used in this study, with higher scores indicating a higher level of irrational beliefs (e.g., “It is very important to me to be loved or approved of by almost everyone I meet”). The internal consistency coefficient for this scale in the current study was 0.76. A one-factor model was built for irrational beliefs, the CFA result showed good model fit (CFI = 0.96, TLI = 0.95), and the absolute index RMSEA was 0.053 with 95%CI [0.047, 0.059] also indicating good fit.

#### Solitude

The solitude behavior scale for short version was used to measure solitude (Liu et al., 2023), which contained four dimensions, including positive solitude (e.g., “I can do the things that I am really interested in when I am alone”), eccentricity (e.g., “I like to stay alone and I am not interested in other people”), social avoidance (e.g., “I feel nervous when talking to someone that I am not familiar with”) and loneliness (e.g., “I feel lonely when no one accompanies with me”). The scale required the participants to indicate their extent of agreement to 16 statements using a 5-point scale ranging from “strongly disagree” to “strongly agree.” A high score indicates a high level of positive solitude, eccentricity, social avoidance and loneliness. The internal consistency coefficient of the four dimensions ranged between 0.83 and 0.86. Confirmatory factor analysis (CFA) was used to examine the factor structure of Solitude. Based on Chen et al. (2012), a four-factor model was examined in this study, the model fit was good (CFI = 0.96, TLI = 0.95), and the absolute index RMSEA was 0.08 with 95% CI [0.080, 0.088] indicating acceptable fit.

#### Mobile phone dependence

Students’ mobile phone dependence was measured with six items selected from Test of Mobile phone Dependence of Vezzoli et al. (2021). The author modified the statements when translating into Chinese. It had two dimensions (e.g., “If I do not have my mobile phone, I feel uneasy”). Three items measured participants’ anxiety without a mobile phone at side and the other three items measured influence of overuse of a mobile phone. A 5-point Likert scale (ranging from 1 = very inappropriate to 5 = very appropriate) was used. A high score indicated a high level of anxiety and overuse. In this study, the internal consistency coefficient of the whole scale was 0.83. For mobile phone use anxiety and mobile phone overuse, the internal consistency coefficients were 0.86 and 0.78 separately. A two-factor structure was examined. The relative model fit was good (CFI = 0.99, TLI = 0.98), and the absolute index RMSEA was 0.06, with 95%CI [0.045, 0.067].

### Data analysis

The factor structure of solitude, irrational beliefs, and mobile phone use was examined using Mplus 8. The following indices were used to determine an acceptable model fit: comparative fit index (CFI > 0.90), Tucker-Lewis index (TLI > 0.90) (Bollen, 1989; Byrne, 2001; Hu and Bentler, 1999), and root mean square error of approximation (RMSEA), values ranging from 0.05 to 0.10 indicate mediocre fit (MacCallum et al., 1996). The bootstrap method (bootstrap = 10,000) was used to calculate the estimates of SEM with 95% confidence interval in this study. If the confidence interval includes 0, which means the coefficient is deemed insignificant. A mediation analysis was conducted with Mplus 8.0 using SEM to model the relationship between irrational beliefs, solitude and mobile phone dependence.

## Results

### Descriptive statistics

The results for solitude, irrational beliefs and mobile dependence among Chinese university students are shown in Table 1. The level of anxiety without mobile phone (*M* = 2.80, *SD* = 0.82) was reported by the participants. Mobile phone overuse (*M* = 2.56, *SD* = 0.78) was lower than the general average of 3. Students scored highest on positive solitude (*M* = 4.11, *SD* = 0.73), then was social avoidance (*M* = 3.12, *SD* = 0.99), and they scored lower on loneliness (*M* = 2.63, *SD* = 0.95) and eccentricity (*M* = 2.23, *SD* = 0.89). Students reported irrational beliefs (*M* = 0.36, *SD* = 0.24) were slightly below the average (0.5).

**Table 1:** Descriptive statistics, alphas, and T-test results for scales and sub-scales.

	Alpha	Mean ± SD			*T*		Mean ± SD		*T*	
		Total	Male	Female		Cohen’s d	City	Rural		Cohen’s d
**Smart phone dependence**										
Mobile phone use anxiety	0.82	2.80 ± 0.82	2.72 ± 0.87	2.97 ± 0.82	−7.14^***^	0.28	2.83 ± 0.88	2.77 ± 0.84	2.03^*^	0.08
Mobile phone overuse	0.78	2.56 ± 0.78	2.53 ± 0.81	2.63 ± 0.76	−3.10^**^	0.12	2.57 ± 0.79	2.56 ± 0.80	0.15	0.01
**Solitude**										
Positive solitude	0.86	4.11 ± 0.73	4.08 ± 0.75	4.19 ± 0.69	−3.12^**^	0.15	4.10 ± 0.75	4.12 ± 0.72	−0.83	0.04
Eccentricity	0.85	2.23 ± 0.89	2.23 ± 0.89	2.22 ± 0.86	0.21	0.01	2.27 ± 0.90	2.19 ± 0.87	2.05^*^	0.09
Social avoidance	0.85	3.12 ± 0.99	3.06 ± 0.99	3.27 ± 0.97	−4.34^***^	0.21	3.14 ± 0.96	3.10 ± 1.01	1.07	0.05
Loneliness	0.83	2.63 ± 0.95	2.60 ± 0.94	2.72 ± 0.96	−2.75	0.13	2.65 ± 0.95	2.61 ± 0.94	1.02	0.04
Irrational beliefs	0.76	0.36 ± 0.24	0.38 ± 0.25	0.32 ± 0.23	4.73^***^	0.22	0.36 ± 0.24	0.36 ± 0.24	0.38	0.02

*p < 0.05; **p < 0.01;***p < 0.001.

A *t*-test indicated that males scored lower on anxiety without mobile (*M* = 2.72, *SD* = 0.87) and mobile phone overuse (*M* = 2.53, *SD* = 0.81) than females (*M* = 2.97, *SD* = 0.82; *M* = 2.63, *SD* = 0.76). Males’ irrational beliefs (*M* = 0.38, *SD* = 0.25) were significantly higher than that of females’ (*M* = 0.32, *SD* = 0.23). Females’ positive solitude (*M* = 4.19, *SD =* 0.69) and social avoidance (*M* = 3.27, *SD =* 0.97) was significantly higher than that of males’ (*M* = 4.08, *SD* = 0.75; *M* = 3.06, *SD* = 0.99). A *t*-test also showed that students from city scored higher on mobile phone use anxiety (*M* = 2.83, *SD* = 0.88) and eccentricity (*M* = 2.27, *SD* = 0.90) than students from rural area (*M* = 2.77, *SD =* 0.84; *M* = 2.19, *SD* = 0.87). Students who were born from only-child family (*M* = 2.32, *SD =* 0.92) scored higher only on eccentricity than those who were not (*M* = 2.21, *SD* = 0.88; *t* = 2.37, *p* < 0.05). No statistically significant differences were found for those from single parent family and those no on other variables.

### SEM results

#### Common method variance

Harman’s (1967) single factor Test was used to test the Common Method Variance. The first common factor explained 17.30% of the total variance, which is below the critical value of 40%. Additionally, the one-factor model, which included all the variables, was tested using CFA. The model fit was very poor (CFI = 0.61, TLI = 0.58, RMSEA = 0.093), indicating no significant issue of common method bias in this study.

#### Direct effects

The study examined the relationship between these two variables by controlling students’ gender and child number in family. The model fit was acceptable (CFI = 0.966, TLI = 0.962, RMSEA = 0.039 with 90% CI [0.038, 0.041]). The results of SEM were summarized in Table 2.

**Table 2:** Standardized coefficient of SEM.

	AMP		OUMP	
	*β*	95%CI	*β*	95%CI
Gender	−0.12^***^ (0.02)	[0.161, −0.085]	−0.04 (0.02)	[−0.074, 0.002]
OCF	0.02 (0.02)	[−0.016, 0.064]	−0.01 (0.02)	[−0.048, 0.035]
Belief	0.09^**^ (0.03)	[0.029, 0.152]	0.12^***^ (0.03)	[0.054, 0.180]
PS	−0.10^***^ (0.03)	[−0.154, −0.036]	−0.11^**^ (0.03)	[−0.169, −0.046]
EC	0.00 (0.03)	[−0.056, 0.062]	0.07^*^ (0.03)	[0.003, 0.131]
SA	0.10^**^ (0.03)	[0.029, 0.173]	0.15^***^ (0.03)	[0.080, 0.224]
LO	0.18^***^ (0.03)	[0.110, 0.255]	0.11^**^ (0.03)	[0.033, 0.181]
Ind by PS	0.02^**^ (0.01)	[0.006, 0.029]	0.02^**^ (0.01)	[0.008, 0.032]
Ind by EC	0.00 (0.01)	[−0.013, 0.015]	0.02^*^ (0.01)	[0.001, 0.033]
Ind by SA	0.02^**^ (0.01)	[0.005, 0.030]	0.02^**^ (0.01)	[0.012, 0.040]
Ind by LO	0.06^***^ (0.01)	[0.034, 0.083]	0.03^**^ (0.01)	[0.011, 0.058]

AMP: Mobile phone use anxiety; OUMP: mobile phone overuse; Gender: female coded as 1, male coded as 0; OCF: family with only one child, “yes” coded as 1, “no” coded as 0; PS: positive solitude; EC: eccentricity; SA: Social avoidance; LO: Loneliness; Ind: Indirect effect.

Females felt more anxious without mobile phone at side than males (*β* = −0.12, 95%CI [−0.161, −0.085]). Males reported less positive solitude (*β* = −0.07, 95%CI [−0.118, −0.023]), social avoidance (*β* = −0.10, 95%CI [−0.147, −0.056]) and loneliness (*β* = −0.07, 95%CI [−0.115, −0.021]) than Females.

Students’ irrational beliefs positively predict anxiety without mobile phone (*β* = 0.09, 95%CI [0.029, 0.152]) and overuse of mobile phone (*β* = 0.12, 95%CI [0.054, 0.180]). Students’ irrational beliefs negatively predict positive solitude (*β* = −0.16, 95%CI [−0.226, −0.098]) while positively predicting eccentricity (*β* = 0.23, 95%CI [0.179, 0.287]), social avoidance (*β* = 0.15, 95%CI [0.096, 0.210]) and loneliness (*β* = 0.31, 95%CI [0.254, 0.363]).

**Figure 2:**
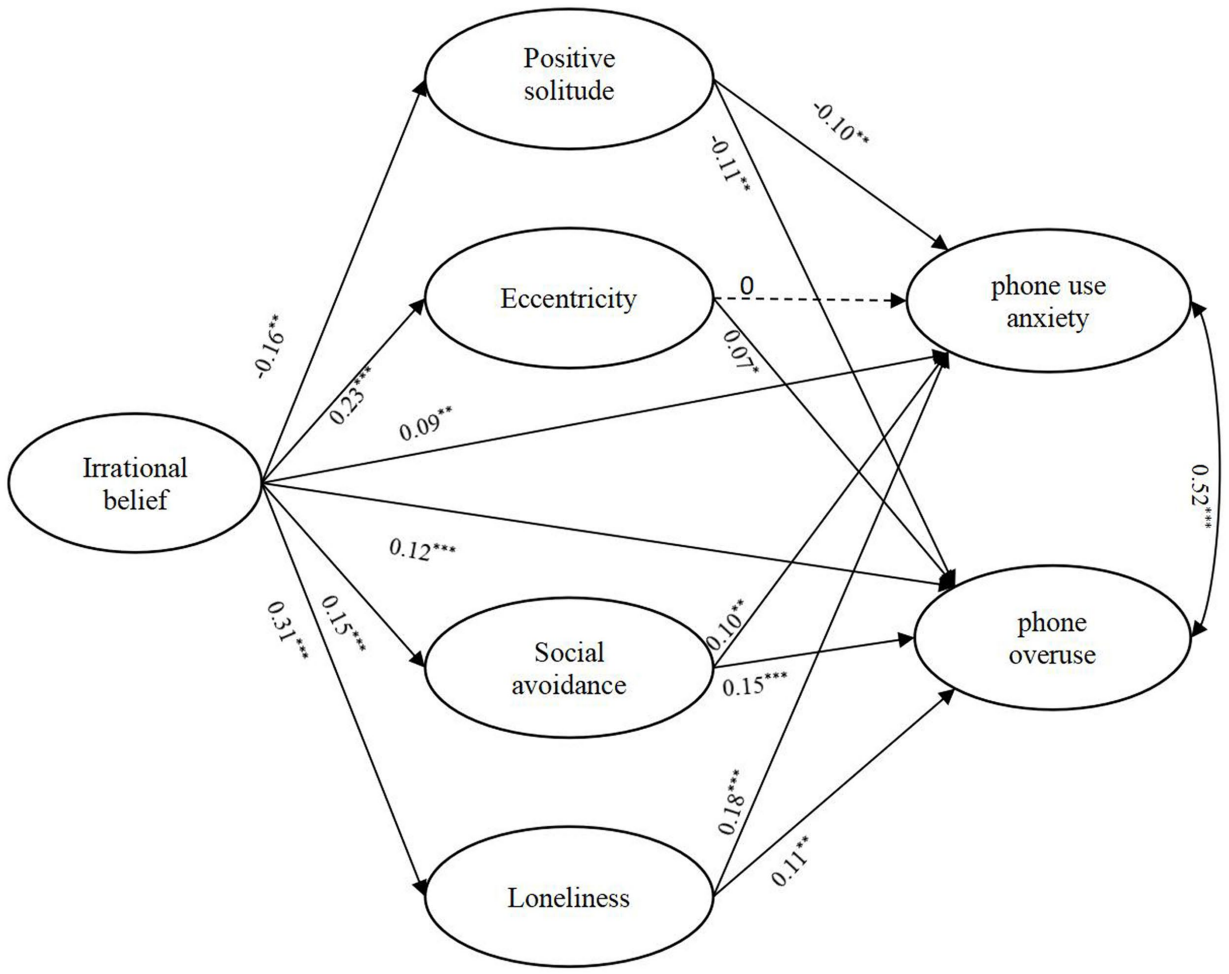
Final model: associations between irrational belief, solitude and mobile phone dependence.

Positive solitude negatively predicts anxious without mobile phone (*β* = −0.10, 95%CI [−0.154, −0.036]) and overuse of mobile phone (*β* = −0.11, 95%CI [−0.169, −0.046]). Social avoidance and loneliness both positively predict anxiety without mobile phone (*β*_sa_ = 0.10, 95%CI [0.029, 0.173]; *β*_lo_ = 0.18, 95%CI [0.110, 0.255]) and overuse of mobile phone (*β_sa_* = 0.15, 95%CI [0.080, 0.224]; *β*_lo_ = 0.11, 95%CI [0.033, 0.181]). Eccentricity positively predict overuse of mobile phone (*β* = 0.07, 95%CI [0.003, 0.131]) (Figure 2).

#### Mediation results

The mediation effect was also examined. Positive solitude mediated the relationship between irrational beliefs and anxiety without mobile phone (*β* = 0.02, 95%CI [0.006, 0.029]) and the relationship between irrational beliefs and mobile phone overuse (*β* = 0.02, 95%CI [0.008, 0.032]). Eccentricity mediated the relationship between irrational beliefs and mobile phone overuse (*β* = 0.02, 95%CI [0.001, 0.033]). Social avoidance mediated the relationship between irrational beliefs and anxiety without mobile phone (*β* = 0.02, 95%CI [0.005, 0.030]) and the relationship between irrational beliefs and mobile phone overuse (*β* = 0.02, 95%CI [0.012, 0.040]). Loneliness mediated the relationship between irrational beliefs and anxiety without mobile phone (*β* = 0.06, 95%CI [0.034, 0.083]) and the relationship between irrational beliefs and mobile phone overuse (*β =* 0.03, 95%CI [0.011, 0.058]).

## Discussion

In the rapid development of technology information and technology era, mobile phones have become the basic necessity for university students. The functionality of mobile phones, while making students’ life more convenient and comfortable, has also led to a new problem, as mobile phone use dependence. This has been the focus of the research reported here. Although previous studies identified various personality traits as significant predictors of mobile phone dependence (Yang et al., 2020), limited attention has been paid to irrational beliefs, and solitude. In this study, we tested the effect of irrational beliefs on mobile phone use dependence and the mediation effect of solitude on the association between these two variables.

Firstly, the gender difference of mobile phone dependence was found. Previous studies showed that males show higher risk of mobile phone addiction (Lee and Kim, 2018; Bisen and Deshpande, 2016). Such as Chen et al. (2017) found male and females preferred different mobile phone applications. Males were more likely to be addicted to game applications, while females were attracted by multimedia and social networking applications. In this study, mobile phone dependence referred to anxiety without mobile phone at side and overuse of mobile phone. The results showed that females were more likely to feel anxious without a mobile phone. This finding is in line with the conclusion of Busch and McCarthy (2021)’s systematic review of 293 studies that females are more prone to problematic mobile phone use. In addition, the study also found that males reported less positive solitude than females, just as Chen et al. (2021) found females score higher on preference for solitude. Females also scored higher on social avoidance and loneliness than males. One of the possible reasons might be that female cared more about the quality of interpersonal relationships (Davcheva and González-Romá, 2022). Thus, they might more sensitive to social relationship and negative aspects of solitude.

Second, irrational beliefs were found to be positively related to mobile phone use dependence (Busch and McCarthy, 2021). Hypothesis 1 was supported. That is, students who scored more highly on irrational beliefs tended to score highly on mobile phone use dependence. Consistent with prior research showing that irrational beliefs are associated with loneliness (Hyland et al., 2019). This study further indicated that irrational beliefs significantly predicted negative aspects of solitude including loneliness, social avoidance and eccentricity. Additionally, one more important finding in the present study was that irrational beliefs were negatively related to positive solitude. In other words, students who scored highly on irrational beliefs also reported high levels of social avoidance and eccentricity and lower levels of positive solitude. Hypothesis 2 was supported.

Third, students’ positive solitude negatively predicted anxiety without a mobile phone and overuse of mobile phone. Students’ social avoidance, loneliness and eccentricity positively predicted anxiety without mobile phone and overuse of mobile phone, those are inconsistent with the finding of Lu et al.’s (2022), who found that positive solitude was significantly positively correlated with nomophobia. There are several possible reasons for this. First, the mobile phone use dependence scales we used in this study was different from theirs; second, they used pearson correlations to compute the association among variables, while in this study, we used structural equation modeling. Given the feature of positive solitude is similar to preference for solitude, our results supported Chen et al. (2021, p. 9)’s finding indicating that a preference for solitude had a negative association with mobile phone addiction.

Fourth, while Chen et al. (2021) showed a direct association between solitude and mobile phone addiction, the current study also showed that solitude played a mediating role in the relationship between irrational beliefs and anxiety without mobile phone and overuse of mobile phone. Irrational beliefs contribute to negative solitude, which in turn leads to increased mobile phone dependence. It suggests that the psycho-social states of Chinese students play an important role in accounting for dependence on mobile phone use. This goes beyond the earlier suggestions that such use was a factor associated with different personality types (Takeo, 2014). As Wang et al. (2022) pointed out “early intervention and identification of those who show signs of PSU (problematic smartphone use) may prevent the development of maladaptive coping responses and addictive behavior, so as to prevent future negative psychosocial consequences” (p. 7). According to the findings of this study, we believed that overt criticism of mobile phone dependence, such as banning or limiting use, may not be dealing with the real problem. Mobile phone dependence is an external behavior, which is the result of rational belief, solitude and other negative psychosocial state. In line with REBT, which emphasizes the role of irrational beliefs in shaping emotional and behavioral responses, it is crucial to address these internal factors. By helping individuals challenge and replace irrational beliefs with more rational and constructive ones, and by addressing feelings of loneliness and other negative psychosocial states, we can more effectively mitigate mobile phone dependence. Guiding students to develop rational beliefs and manage their loneliness is a more effective approach to preventing mobile phone dependence among university students.

## Limitation and future direction

This study has not directly addressed the issue of dependence on the relationship between dependence on mobile phone use and student academic development and achievement. Yet Wang et al. (2022) and Li et al. (2021) have suggested that such links exist. Given the results of this study, these links need to be explored further. The focus of such research would not necessarily be mobile phone dependence itself, but rather the psychological states of students who display such dependence. There is a considerable literature on the correlates of psychosocial conditions and learning, although much of it has been focused on secondary school students. What is needed now is a better understanding of how and why such conditions, both negative and positive, influence university students, and what is the effect on their academic learning.

This study faces certain limitations of this study that provide some directions for future studies. First, the methodology used in this study were self-report measures, which may have caused the finding to be limited by the structure of the scale. Future research should also include qualitative methods (interviews, etc.) to investigate the interrelationship among irrational beliefs, solitude and mobile phone use dependence to provide a deeper understanding of the influence mechanism based on college students’ personal experience and a discourse analysis methodology. Second, considering the sociocultural characteristics of solitude, comparison with students in other counties would be valuable. Future work should examine contextual factors to highlight more practical implications.

## Data Availability

The raw data supporting the conclusions of this article will be made available by the authors, without undue reservation.
